# From Inclusion Complexes to Metabolic Signaling: The Emerging Role of γ-Cyclodextrin in Gut Microbiota and Metabolic Regulation

**DOI:** 10.3390/molecules31142415

**Published:** 2026-07-09

**Authors:** Pirscoveanu Denisa Floriana Vasilica, Pluta Ion Dorin, Dîrnu Rodica, Carmen Vladulescu, Diana-Maria Trasca, Renata Maria Varut, Adina Kamal, Maria Stoica, Gabriela Pura, Romeo Popa, Virginia Radulescu, George Alin Stoica

**Affiliations:** 1 Department of Neurology, Faculty of Medicine, University of Medicine and Pharmacy of Craiova, 200349 Craiova, Romania; denisa.pirscoveanu@umfcv.ro; 2 Faculty of Medical and Behavioral Sciences, Constantin Brâncuși University of Târgu Jiu, 210185 Târgu Jiu, Romania; dorin.pluta@e-ucb.ro (P.I.D.); rodica.dirnu@e-ucb.ro (D.R.); 3 Department of Biology and Environmental Engineering, Faculty of Horticulture, University of Craiova, 200585 Craiova, Romania; carmen.vladulescu@edu.ucv.ro; 4 Department of Internal Medicine, University of Medicine and Pharmacy of Craiova, 200349 Craiova, Romania; adina.kamal@umfcv.ro; 5 Research Methodology Department, Faculty of Pharmacy, University of Medicine and Pharmacy of Craiova, 200349 Craiova, Romania; 6 Department of Intensive Care and Anesthesia, Emergency County Hospital, 200349 Craiova, Romania; maria.stoica@umfcv.ro; 7 Department of Medical Devices and Pharmaceutical Practice, Iuliu Hațieganu University of Medicine and Pharmacy, 400012 Cluj-Napoca, Romania; gabrielapura@gmail.com; 8 Department of Pharmacology, University of Medicine and Pharmacy of Craiova, 200349 Craiova, Romania; romeo.popa@umfcv.ro; 9 Department of Nephrology, Faculty of Medicine, University of Medicine and Pharmacy of Craiova, 200349 Craiova, Romania; virginia.radulescu@umfcv.ro; 10 Department of Pediatric Surgery, Faculty of Medicine, University of Medicine and Pharmacy of Craiova, 200349 Craiova, Romania; alin.stoica@umfcv.ro

**Keywords:** γ-cyclodextrin, gut microbiota, short-chain fatty acids, obesity, type 2 diabetes, lipid metabolism, prebiotic effects, metabolic health, glycemic control

## Abstract

γ-Cyclodextrin (γ-CD) is a cyclic oligosaccharide with high aqueous solubility, low toxicity, and a large internal cavity that enables inclusion complex formation with selected bioactive compounds. Beyond its established role as a pharmaceutical and food excipient, emerging evidence suggests that γ-CD may influence metabolic regulation through interactions with the gut microbiota, microbial fermentation products, and host metabolic signaling pathways. This review synthesizes current evidence on the effects of γ-CD on short-chain fatty acid production, lipid homeostasis, glycemic control, and obesity- and type 2 diabetes-related metabolic disturbances. Particular attention is given to the gut–metabolism axis, SCFA-mediated GPCR signaling, microbial taxa potentially involved in γ-CD fermentation, and the relative contribution of prebiotic-like effects versus lipid-binding mechanisms. Available data indicate that γ-CD may modulate microbial composition and metabolic outcomes, but most evidence derives from in vitro experiments, animal models, and limited human studies. Therefore, the clinical relevance of γ-CD remains insufficiently established. Future studies should include well-designed human trials, standardized doses, multi-omics analyses, and direct comparisons between native and modified cyclodextrins to clarify whether γ-CD can be translated into nutritional or therapeutic strategies for metabolic disorders.

## 1. Introduction

**Figure 1:**
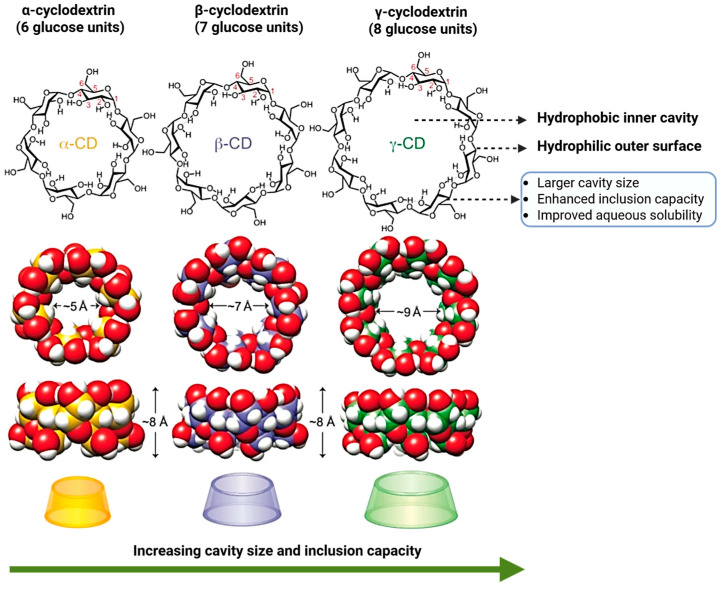
Structural comparison of α-, β-, and γ-CDs, highlighting differences in cavity size, molecular conformation, and inclusion capacity. Created in BioRender. Varut, M. (2026) https://BioRender.com/lt4tx8o (accessed on 30 June 2026).

Cyclodextrins (CDs) represent a group of cyclic oligosaccharides formed from D-glucose units connected through α-(1→4) glycosidic linkages [1,2]. Structurally, they are arranged as macrocyclic molecules with a characteristic torus-like three-dimensional configuration. The size of the internal cavity depends on the number of glucose residues that make up the ring. The most widely studied forms include α-, β-, and γ-CDs, consisting of six, seven, and eight glucopyranose units, respectively. Among the naturally occurring cyclodextrins, γ-CD is distinguished by its larger ring structure, consisting of eight glucose units, compared to six and seven in α- and β-cyclodextrin, respectively. The structural differences between α-, β-, and γ-CDs, particularly regarding cavity size and inclusion capacity, are illustrated in Figure 1.

This increased number of glucose subunits results in a wider internal cavity, which enhances the ability of γ-CD to accommodate bulkier guest molecules and form stable inclusion complexes. In addition, γ-CD has a higher molecular weight and a larger external diameter than α- and β-CD, contributing to differences in aqueous solubility, cavity dimensions, and interaction with guest compounds. These structural characteristics make γ-CD particularly suitable for applications involving the encapsulation, stabilization, and delivery of larger or more complex bioactive substances [3].

In addition to conventional inclusion complexes, cyclodextrins can also be used as building blocks for cross-linked polymeric systems known as cyclodextrin nanosponges. These supramolecular networks are able to entrap, stabilize, and release bioactive molecules in a controlled manner, thereby expanding the pharmaceutical and biomedical applications of cyclodextrin-based delivery systems. Although the present review focuses primarily on native γ-CD and its metabolic relevance, CD-nanosponges represent an important technological extension of cyclodextrin chemistry and should be considered within the broader field of advanced CD-based formulations [4].

Among the three native cyclodextrins, γ-CD demonstrates several advantageous properties when compared with its α- and β-counterparts. In terms of aqueous solubility at room temperature, γ-CD exhibits a markedly higher value, approximately 23.2 g/100 mL, compared with α-cyclodextrin, approximately 14.5 g/100 mL, and especially β-cyclodextrin, which has a much lower solubility, approximately 1.8 g/100 mL [5,6,7,8]. This improved solubility contributes to the broader use of γ-CD in drug delivery systems, nutritional applications, and the food industry, where efficient dispersion, inclusion complex formation, and improved bioavailability of guest molecules are essential [5].

From a safety perspective, γ-CD is characterized by low toxicity, with reported LD_50_ values exceeding 3750 mg/kg (IV, rat), indicating a favorable toxicological profile [5]. In contrast, β-cyclodextrin has been associated with moderate toxicity, including nephrotoxic effects at lower doses (LD_50_ ≈ 788 mg/kg, IV, rat), which limits its systemic use despite its utility in pharmaceutical and environmental applications [7,8]. Similarly, while α-cyclodextrin also presents low toxicity (LD_50_ ≈ 1000 mg/kg, IV, rat), its comparatively smaller cavity size restricts its ability to encapsulate larger molecules, thereby narrowing its functional scope in advanced delivery systems [6].

Overall, the combination of higher solubility, lower toxicity, and a larger cavity structure positions γ-CD as a particularly versatile candidate for applications requiring the inclusion and stabilization of complex or bulky bioactive compounds, especially within pharmaceutical and nutritional contexts [5].

γ-CD is produced from starch via enzymatic processes and is distinguished by its characteristic molecular architecture, consisting of a relatively hydrophobic inner cavity surrounded by a hydrophilic outer surface [1,2,9]. This amphiphilic configuration allows γ-CD to encapsulate a wide range of guest molecules through inclusion complex formation, which underlies its broad applicability across multiple scientific fields, including pharmaceutical sciences, biotechnology, environmental applications, and materials engineering [10,11,12].

A key feature of γ-CD is its capacity to improve the physicochemical properties of bioactive compounds, particularly by increasing their aqueous solubility, chemical stability, and overall bioavailability. These properties have contributed significantly to its growing role in drug delivery strategies, where inclusion complexes can enhance the therapeutic performance of various active pharmaceutical ingredients [13,14]. Furthermore, structural modification of cyclodextrins has enabled the development of derivatives with optimized characteristics, allowing more precise adaptation to specific formulation requirements.

Although γ-CD has applications in several technological fields, the present review focuses only on those properties with direct nutritional or biomedical relevance, particularly solubility, safety, inclusion complex formation, intestinal availability, microbial fermentation, and potential effects on metabolic regulation [15,16].

The relevance of γ-CD to metabolic regulation does not derive solely from its chemical structure but from the way its physicochemical properties may translate into biological effects. Its larger cavity size and high aqueous solubility may influence the inclusion and intestinal availability of lipophilic compounds, while its partial resistance to upper gastrointestinal digestion may allow a fraction of γ-CD or γ-CD-containing complexes to reach the colon, where microbial fermentation can occur. Through these mechanisms, γ-CD may connect cyclodextrin chemistry with gut microbiota modulation, SCFA production, lipid handling, and host metabolic signaling. Metabolic health refers to the coordinated regulation of glucose concentrations, lipid profiles, blood pressure, adiposity, and inflammatory status, maintained through efficient nutrient utilization and metabolic flexibility [17,18,19]. Disturbances in these processes have contributed to the increasing prevalence of metabolic dysfunction, obesity, type 2 diabetes, cardiovascular disease, and metabolic dysfunction-associated fatty liver disease, conditions strongly influenced by lifestyle, dietary quality, and gut microbiota composition [20,21,22,23,24].

Because diet and intestinal microbiota play central roles in metabolic regulation, dietary components capable of modulating microbial fermentation, short-chain fatty acid (SCFA) production, intestinal barrier function, and lipid handling have become increasingly relevant in metabolic research [25,26,27,28,29,30]. Within this framework, γ-CD is of interest because it may act at the interface between dietary carbohydrate-like structures, gut microbial metabolism, and host metabolic regulation. Beyond its conventional role as a formulation excipient, γ-CD may contribute to metabolic regulation through interconnected mechanisms, including modulation of fermentative microbial taxa, stimulation of SCFA production, and alteration of lipid or bioactive compound availability via inclusion complex formation. These properties support its consideration as a potential dietary modulator of the gut–metabolism axis [31,32].

From a nutritional and regulatory perspective, cyclodextrins differ across regions. In the European Union, β-cyclodextrin is approved as a food additive (E459), with an acceptable daily intake (ADI) of 5 mg/kg body weight per day [33]. By contrast, α- and γ-CD are classified as novel food ingredients, and specific ADI values have not been formally established [34].

At the international level, the Joint FAO/WHO Expert Committee on Food Additives (JECFA) has recognized cyclodextrins as General Food Standard Additives (GFSA). While an intake limit has been established for β-cyclodextrin (0–5 mg/kg body weight per day), no such restriction has been imposed for α- or γ-CD, indicating a favorable safety profile and broader flexibility in their use within food products [35,36,37]. Similarly, regulatory authorities in the United States, including the Food and Drug Administration (FDA), classify native cyclodextrins as Generally Recognized as Safe (GRAS). Extensive toxicological evaluations, particularly for α- and γ-CD, have not identified significant safety concerns, further supporting their application in nutritional contexts [38].

In light of these considerations, the present review aims to explore the role of γ-CD in the context of metabolic health and nutrition. By examining its structural characteristics, functional properties, and potential physiological effects, this work seeks to highlight its relevance as a dietary component with possible benefits in modulating key metabolic processes (Table 1).

## 2. Metabolic Health, Gut Microbiota, and Major Metabolic Disorders

The human gut microbiota comprises a highly diverse and dynamic community of microorganisms—including bacteria, archaea, fungi, and viruses—that colonize the gastrointestinal tract and maintain a symbiotic relationship with the host [44]. Owing to its extensive contribution to physiological regulation, immune homeostasis, and metabolic function, the gut microbiota is frequently regarded as an “overlooked organ” [45]. Its composition is continuously shaped by environmental and host-related factors, including nutrient availability, pH, bile acid composition, oxygen tension, and other physicochemical conditions within the intestinal milieu [45]. Although microbial composition varies along the gastrointestinal tract, the colon harbors the highest density and diversity of microorganisms, with Firmicutes, Bacteroidetes, and Proteobacteria accounting for the majority of the gut microbial population [46,47].

From a functional perspective, the gut microbiota is deeply involved in carbohydrate and protein metabolism, energy harvesting, synthesis of essential metabolites, immune modulation, maintenance of intestinal barrier integrity, drug metabolism, and xenobiotic biotransformation [48,49,50,51]. Despite pronounced interindividual variability, healthy microbiota are generally characterized by high diversity, functional stability, and a conserved repertoire of core metabolic activities [48,52,53,54]. Consequently, perturbations in this ecological balance may profoundly influence host metabolism and contribute to the pathogenesis of chronic metabolic disorders.

A disrupted microbial state, commonly referred to as gut dysbiosis, has been closely associated with oxidative stress, inflammation, and metabolic dysfunction [55]. Dysbiosis may impair intestinal barrier function, alter microbial metabolite production, and trigger immune activation, thereby promoting the generation of reactive oxygen species (ROS) and perpetuating a vicious cycle between oxidative stress and microbial imbalance [56,57].

**Figure 2:**
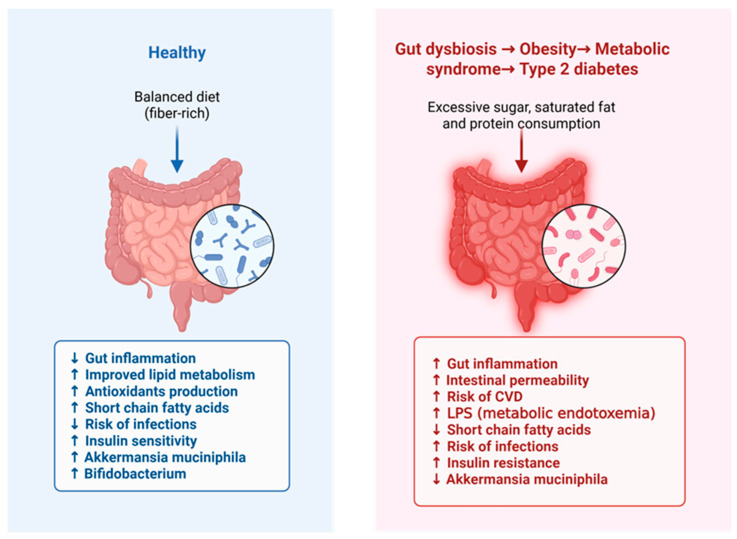
Healthy vs. dysbiotic gut microbiota and their metabolic effects, highlighting the role of diet, SCFA production, and inflammation in metabolic disorders. CVD, cardiovascular disease; LPS, lipopolysaccharide. Upward arrows (↑) indicate an increase, whereas downward arrows (↓) indicate a decrease in the respective parameter. Created in BioRender. Varut, M. (2026) https://BioRender.com/idgfww4 (accessed on 30 June 2026).

The differences between healthy gut microbiota and dysbiosis, as well as their metabolic consequences, are illustrated in Figure 2.

Experimental evidence indicates that excessive ROS production may itself alter microbial diversity and reshape gut community structure [57], whereas interventions aimed at restoring eubiosis, including probiotics and bioactive dietary compounds, can attenuate oxidative stress and inflammatory signaling [58,59,60]. At the same time, ROS should not be viewed exclusively as harmful molecules, as they also participate in physiological signaling pathways that support epithelial renewal and intestinal homeostasis [61].

Among the most important functional outputs of the gut microbiota are short-chain fatty acids (SCFAs), which are generated through the bacterial fermentation of indigestible dietary substrates, especially plant-derived fibers [62,63]. These metabolites arise from the breakdown of polysaccharides, oligosaccharides, glycoproteins, peptides, and other undigested substrates by anaerobic microorganisms [64,65,66,67]. The principal SCFAs—acetate, propionate, and butyrate—are the dominant end-products of this fermentation process [68,69], and, once absorbed, they exert pleiotropic effects on local and systemic metabolism [70,71,72]. Many of the recognized benefits of dietary fiber and prebiotic interventions are thought to be mediated, at least in part, through SCFA production [73,74,75,76]. In physiological conditions, acetate, propionate, and butyrate are present in an approximate molar ratio of 60:20:20 [77].

SCFAs are rapidly absorbed throughout the colon and subsequently metabolized in the intestinal epithelium, liver, and peripheral tissues [69,78]. Butyrate serves as the principal energy substrate for colonocytes [79], whereas propionate and butyrate contribute to hepatic metabolic pathways, including gluconeogenesis [80], and acetate may enter the systemic circulation to support energy production in peripheral tissues such as skeletal muscle [81,82]. Beyond their energetic role, SCFAs also act as signaling molecules through specific G protein-coupled receptors, mainly, GPR41/FFAR3, GPR43/FFAR2, GPR109A/HCAR2, and, in experimental models, Olfr78. Through these receptors, acetate, propionate, and butyrate can influence glucose homeostasis, lipid metabolism, enteroendocrine hormone secretion, inflammatory pathways, immune responses, and vascular regulation [83,84,85,86,87]. Collectively, these observations position the gut microbiota–SCFA axis as a central mediator of metabolic health.

Obesity, one of the most prevalent metabolic disorders worldwide, is characterized by excessive fat accumulation and represents a major risk factor for T2DM, cardiovascular disease, metabolic-associated liver disorders, and several malignancies [88,89,90,91,92,93,94,95,96,97,98,99,100]. It generally arises from a chronic imbalance between energy intake and expenditure, leading to excess storage of lipids in adipose tissue and ectopic depots [101,102,103]. Adipose tissue is now widely recognized as an active endocrine organ that regulates systemic metabolism through the secretion of adipokines, cytokines, and extracellular mediators [104,105,106,107,108,109,110,111,112,113]. Excess adiposity is accompanied by adipose tissue hypertrophy and hyperplasia, immune cell infiltration, and persistent low-grade inflammation, all of which contribute to insulin resistance, dyslipidemia, and broader metabolic impairment [108,109,110,111,112,113,114,115]. In recent years, the gut microbiota has emerged as a major environmental determinant of obesity and obesity-related metabolic complications [116,117,118,119,120,121,122,123,124,125]. Both animal and human studies have shown that microbial composition can influence dietary energy extraction, adiposity, and host metabolic efficiency [126,127,128,129,130,131,132]. Although early studies proposed an increased Firmicutes/Bacteroidetes ratio as a microbial feature of obesity, later investigations produced inconsistent results. Therefore, this ratio should not be considered a universal or reliable biomarker of obesity. Obesity-associated dysbiosis is better interpreted as a complex, context-dependent microbial and functional alteration involving microbial diversity, SCFA-producing taxa, intestinal barrier function, bile acid metabolism, and inflammatory signaling [120,122,133,134,135,136]. Experimental studies in germ-free and conventionalized animals further demonstrated that an “obese microbiome” can promote fat accumulation, alter lipoprotein lipase activity, and increase energy harvest from the diet [116,117,119,137]. Dysbiosis may also contribute to obesity through increased intestinal permeability, metabolic endotoxemia, chronic inflammation, and disruption of gut–brain–adipose signaling pathways [138,139,140,141,142]. Within this context, SCFAs have attracted particular attention as mediators of host energy balance and adiposity regulation [143,144,145,146,147,148,149,150,151]. However, the relationship between SCFA levels and obesity in humans remains heterogeneous, with studies reporting both positive and inverse associations between fecal or circulating SCFAs and anthropometric indices such as BMI, waist circumference, and visceral adiposity [152,153,154,155,156,157,158,159,160,161,162].

Obesity and T2DM should be viewed as systemic, multi-organ disorders rather than isolated disturbances of adipose tissue or pancreatic β-cell function. Chronic nutrient excess promotes adipocyte hypertrophy, hypoxia, macrophage infiltration, and secretion of pro-inflammatory cytokines, generating a persistent low-grade inflammatory state commonly referred to as metaflammation. This inflammatory milieu contributes to insulin resistance in adipose tissue, skeletal muscle, and liver, while increased lipolysis and ectopic lipid deposition further impair hepatic glucose production and lipid handling. In parallel, pancreatic β-cells are exposed to glucotoxicity, lipotoxicity, oxidative stress, and inflammatory mediators, which progressively compromise insulin secretion. The gut microbiota participates in this network by modulating intestinal permeability, metabolic endotoxemia, SCFA production, bile acid signaling, and gut–brain communication. Therefore, the progression from obesity to T2DM involves reciprocal interactions between immune cells, adipose tissue, liver, pancreas, brain, and gut, providing a mechanistic rationale for studying γ-CD within the gut–metabolism axis.

T2DM is the most common form of diabetes, accounting for approximately 90% of all cases, and is characterized by insulin resistance, pancreatic β-cell dysfunction, impaired glucose utilization, and multiple metabolic abnormalities involving lipid and amino acid handling [163,164,165,166,167,168,169,170]. Its development is strongly associated with obesity and dietary excess in many populations [171,172,173,174,175], although substantial heterogeneity exists across ethnic and geographic groups, indicating that T2DM cannot be reduced to a single phenotypic or genetic pattern [176,177,178,179]. Despite major therapeutic advances, T2DM remains a leading cause of cardiovascular morbidity, blindness, renal failure, neuropathy, impaired wound healing, and premature mortality [180,181,182,183,184,185,186,187,188,189,190]. Dietary patterns play a fundamental role in disease risk and progression, with high consumption of sugars, sweetened beverages, processed foods, and energy-dense diets being associated with greater metabolic risk, whereas plant-rich and fiber-rich dietary patterns appear protective [191,192,193,194,195,196,197,198,199,200,201,202].

The microbiota–SCFA axis has also been increasingly implicated in diabetes pathophysiology. SCFAs may exert protective effects through GPCR-mediated signaling in enteroendocrine cells and pancreatic β-cells, thereby contributing to glucose regulation and metabolic homeostasis [203]. Experimental studies suggest that higher SCFA availability is associated with improved immune regulation, reduced diabetes susceptibility, and enhanced metabolic resilience [204,205,206]. These effects include promotion of regulatory T-cell differentiation, modulation of inflammatory pathways, and epigenetic regulation of host responses [204,205,206,207,208]. In parallel, obesity-related metabolic derangements—including lipotoxicity, chronic inflammation, altered adipokine signaling, and β-cell stress—create a mechanistic bridge between excess adiposity and T2DM development [209,210,211,212,213,214,215,216,217,218,219,220,221,222,223,224,225,226,227,228,229,230,231,232].

Alterations in gut microbial composition have been consistently reported in T2DM, although no single universal microbial signature has been established [233,234,235,236,237,238,239,240,241,242]. Reduced microbial diversity is a recurrent finding [237,238,239], alongside depletion of beneficial butyrate-producing bacteria such as *Faecalibacterium prausnitzii*, *Roseburia intestinalis*, *Roseburia inulinivorans*, *Eubacterium rectale*, *Ruminococcus*, and *Subdoligranulum* [234,243]. Conversely, opportunistic pathogens and taxa associated with metabolic imbalance—including *Bacteroides caccae*, *Clostridium hathewayi*, *Clostridium ramosum*, *Clostridium symbiosum*, *Eggerthella lenta*, *Escherichia coli*, *Blautia*, *Coprococcus*, *Peptostreptococcus*, *Parasutterella*, and *Collinsella*—have been reported at increased abundance in T2DM cohorts [234,241,242,243]. Reduced abundance of *Bifidobacterium*, *Bacteroides*, and *Prevotella* has also been observed [244,245], whereas lower levels of *Akkermansia muciniphila* and *Faecalibacterium prausnitzii* appear particularly relevant, given their association with intestinal barrier integrity, anti-inflammatory effects, and improved insulin sensitivity [241,243,246,247,248,249,250,251]. Similar patterns have also been described in prediabetes, suggesting that dysbiosis may precede overt disease and may eventually provide clinically useful biomarkers of metabolic risk (Table 2) [252].

### Dietary Intervention on Gut and T2DM

A growing body of evidence indicates that dietary interventions can significantly modulate gut microbiota composition and diversity in patients with T2DM. Several studies have reported changes in microbial diversity and richness following dietary modification. For instance, a multicenter randomized clinical trial in prediabetic overweight adults demonstrated that a low-energy diet significantly altered gut microbial diversity, with significant changes in alpha-diversity indices and community structure based on beta-diversity distance metrics, together with increased bacterial richness [253]. Similarly, both low-carbohydrate almond-based and low-fat diets were shown to increase alpha diversity in T2DM patients, although without significant differences in beta diversity between groups [254]. Fiber-rich dietary patterns, such as the Ma-Pi 2 diet, also promoted favorable trends in microbial diversity over time [255], whereas Mediterranean diet interventions yielded more heterogeneous results, with modest or non-significant changes in diversity but occasional increases in bacterial richness or shifts in community structure [256,257].

Beyond diversity metrics, dietary composition exerts a pronounced influence on specific microbial taxa. High-fiber diets consistently promote the growth of beneficial, SCFA-producing bacteria, including *Faecalibacterium prausnitzii*, *Lachnospiraceae*, *Akkermansia muciniphila*, *Bifidobacterium longum*, and *Bacteroides fragilis*, while reducing opportunistic or metabolically unfavorable species [255,258,259,260]. Functional food interventions further reinforce these effects, notably increasing *Akkermansia muciniphila* and *Faecalibacterium prausnitzii* abundance and reducing Prevotella copri levels [260].

Dietary interventions also induce compositional shifts at higher taxonomic levels. Low-energy diets have been associated with increased relative abundance of Verrucomicrobia and Bacteroidetes, alongside reductions in Actinobacteria and Firmicutes [253]. At the genus level, increases in Akkermansia, Ruminococcus, and Bacteroides have been observed, although some studies report concomitant decreases in other beneficial taxa, including Faecalibacterium and Bifidobacterium [253]. Mediterranean diet interventions have been linked to shifts in microbial ratios, such as increased Prevotella/Bacteroides and Firmicutes/Bacteroidetes proportions over time [256].

Distinct dietary patterns produce differential microbiota responses. Ketogenic diets have been shown to increase taxa such as Akkermansia and Eubacterium while reducing Firmicutes and Actinobacteria, whereas Mediterranean diets tend to favor Firmicutes and Actinobacteria expansion [257]. Low-carbohydrate diets may promote SCFA-producing genera such as Ruminococcus, Roseburia, and Eubacterium [254], while amino acid-restricted diets can significantly alter phylum-level distributions, including increased Bacteroidetes and decreased Firmicutes [261]. Personalized dietary interventions appear to exert broader and more individualized effects on microbiota composition compared to standardized dietary patterns [262,263].

Collectively, these findings highlight that dietary modulation of the gut microbiota in T2DM is highly dependent on macronutrient composition and dietary quality. High-fiber dietary patterns consistently support beneficial microbial taxa and SCFA production, whereas high-fat or low-carbohydrate diets induce distinct, sometimes less favorable, microbial shifts. These alterations underscore the central role of diet–microbiota interactions in metabolic regulation and T2DM management (Table 3). Although most dietary interventions summarized in Table 3 do not directly involve γ-CD, they are included to provide metabolic and microbiota-related context. These studies show that diet-induced modulation of gut microbiota, SCFA production, and glycemic control is clinically relevant in T2DM. This background is important because γ-CD is discussed here as a potential dietary modulator of the gut–metabolism axis rather than as an established antidiabetic therapy.

## 3. γ-CD on Gut Microbiota and SCFA

**Figure 3:**
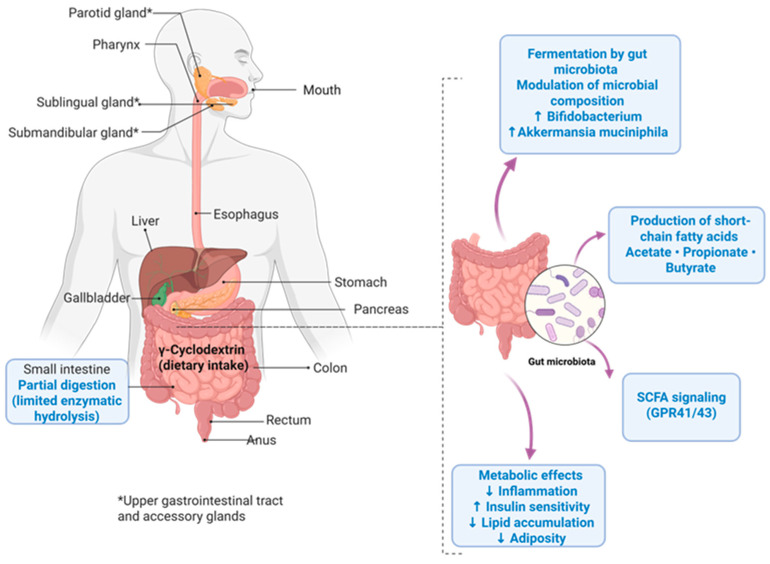
Proposed mechanisms linking γ-CD to metabolic regulation. γ-CD may reach the colon partially undigested or as inclusion complexes, where it can be fermented by gut microbiota and promote SCFA production. SCFAs may activate GPCR-dependent signaling pathways, including GPR41, GPR43, and GPR109A, and may also influence host metabolism through HDAC inhibition, enteroendocrine hormone release, improved barrier function, reduced metabolic endotoxemia, and attenuation of low-grade inflammation. In parallel, γ-CD may indirectly influence lipid and glucose metabolism through inclusion complex formation and AMPK-related pathways, although native γ-CD appears to have limited direct cholesterol-sequestering activity. Abbreviations: SCFA, short-chain fatty acids. Upward arrows (↑) indicate an increase, whereas downward arrows (↓) indicate a decrease in the indicated parameter or bacterial abundance. Created in BioRender. Varut, M. (2026) https://BioRender.com/oylpip8 (accessed on 30 June 2026).

Wupper et al. [264] reported in a clinical trial that cyclodextrin supplementation enhanced endurance exercise performance in both mice and human males. Supplementation with cyclodextrins increased the abundance of *Bacteroides uniformis* and improved exercise performance in human males, suggesting a role for cyclodextrins in modulating gut microbiota to enhance physical capacity, beyond classical prebiotic functions. The mechanisms underlying the metabolic effects of γ-CD, including its interaction with the gut microbiota and subsequent SCFA production, are illustrated in Figure 3.

Diets supplemented with cyclodextrins, including γ-CD, and oligofructose-enriched inulin increased the *Bacteroidetes/Firmicutes* ratio compared to a high-fat Western diet. A higher ratio of these strains was associated with improved health outcomes [265]. *Proteobacteria* were particularly abundant when cyclodextrins were included in the diet.

Ren et al. [266] demonstrated that male C57BL/6J mice on a high-fat diet supplemented with 5.5% cyclodextrin gained less weight and adipose tissue than mice on a high-fat diet without supplementation. Cyclodextrin intake increased beneficial gut bacteria such as *Bacteroides*, *Bifidobacterium*, and *Lactobacillus*, which are typically reduced by high-fat feeding. Additionally, cyclodextrin supplementation enhanced the production of organic acids and SCFAs, promoting favorable lipid metabolism and anti-obesity effects.

Guevara et al. [267] investigated cyclodextrin supplementation in dogs, evaluating in vitro fermentation, nutrient digestibility, fecal microbiota, and serum lipid profiles. Diets containing varying amounts of cyclodextrins showed that nutrient absorption in the small intestine and overall fecal microbiota were not significantly altered, yet total tract nutrient digestibility and fecal dry matter decreased, indicating modified large-intestine fermentation. In vitro fermentation experiments showed that cyclodextrin type influenced SCFA production: γ-CD led to the highest total SCFA and acetate production as well as the greatest butyrate generation; β-cyclodextrin produced high total SCFA and acetate; and α-cyclodextrin primarily increased propionate, with lower SCFA and acetate levels and longer times to reach peak production [267,268] (Table 4).

SCFAs present in the cecum are primarily produced by gut bacteria fermenting undigested carbohydrates. These microbial metabolites play crucial roles in maintaining host physiological functions. Consumption of a high-fat diet was associated with a marked reduction in total SCFA and acetate levels in cecal contents, while butyrate (*p* > 0.05) and valerate (*p* < 0.05) levels were slightly elevated.

Supplementation with cyclodextrins, particularly γ-CD, significantly enhanced total SCFA concentrations in the cecum of mice receiving a high-fat diet. Inclusion of 6% γ-CD resulted in nearly a 1.44-fold increase in SCFA levels, showing a more pronounced effect than cellulose. This SCFA-promoting activity was largely driven by increases in acetate and propionate [268].

γ-CD supplementation was also found to reduce isobutyrate levels, while leaving butyrate concentrations largely unchanged [269]. In addition to the effects of native γ-CD on microbial fermentation and SCFA production, hydroxypropyl-γ-cyclodextrin (HP-γ-CD) has also been evaluated for its influence on the growth of selected probiotic and lactic acid bacterial strains. These findings are summarized in Table 4 and indicate that HP-γ-CD may differentially support the growth of specific bacterial species, although these data should be interpreted as strain-specific and not as a complete representation of the human gut microbiota.

**Table 4:** Effects of HP-γ-CD on selected probiotic and lactic acid bacterial strains.

Bacterial Strain	HP-γ-CD Effect
*Lactobacillus acidophilus*	↑
*Lactobacillus casei*	→
*Lactobacillus plantarum*	↑
*Lactobacillus brevis*	↑
*Lactobacillus rhamnosus GG*	→
*Lactobacillus reuteri*	→
*Pediococcus pentosaceus*	↑
*Lactococcus lactis*	↑
*Lactobacillus fermentum*	↑
*Streptococcus thermophilus*	↑

↑ = increase in bacterial growth; → = no significant change [270].

Taken together, the metabolic effects of γ-CD are unlikely to result from a single mechanism. Current evidence suggests a combined model in which γ-CD may exert prebiotic-like effects by supporting microbial fermentation and SCFA production, while also modifying the intestinal availability of selected lipids or bioactive compounds through inclusion complex formation. However, native γ-CD appears to have limited direct cholesterol-sequestering capacity compared with some modified cyclodextrins. Therefore, the most plausible mechanism involves indirect microbiota-mediated and metabolite-mediated effects, rather than simple lipid sequestration alone.

### 3.1. Indirect Effects of γ-Cyclodextrin on Lipid Homeostasis: Mechanistic Insights and Metabolic Implications

For clarity, the evidence discussed in this subsection should be interpreted according to the specific form of γ-CD investigated. Native γ-CD, chemically modified γ-CD derivatives, and γ-CD-containing inclusion complexes may differ substantially in solubility, binding affinity, lipid interaction, intestinal behavior, and biological effects. Therefore, effects observed with modified derivatives or γ-CD-based formulations should not be automatically attributed to native γ-CD.

Regarding native γ-CD, available evidence suggests that its direct capacity to sequester dietary cholesterol is limited. Experimental data indicate that native γ-CD exhibits minimal ability to form inclusion complexes with cholesterol, with negligible cholesterol solubilization across a range of concentrations. Consequently, its capacity to reduce intestinal cholesterol absorption appears weaker than that of some modified cyclodextrins. This limited interaction is probably related to structural specificity, including the larger cavity size and lower binding affinity of native γ-CD for sterol molecules. Thus, the potential effects of native γ-CD on lipid homeostasis are more likely to occur through indirect mechanisms, such as microbial fermentation, SCFA production, modulation of intestinal metabolites, or altered availability of selected lipophilic compounds, rather than through direct cholesterol sequestration alone [271].

Modified γ-CD derivatives may exert different effects on intracellular lipid handling. For example, hydroxypropyl-γ-cyclodextrin (HPγCD) has been investigated in Niemann–Pick type C models, a disorder characterized by impaired lysosomal cholesterol trafficking. HPγCD treatment was associated with reduced intracellular cholesterol accumulation and increased expression of lysosome-associated membrane protein 1 (LAMP-1), a key protein involved in lysosomal function and cholesterol mobilization. These findings suggest that modified γ-CD derivatives may facilitate cholesterol export from late endosomal/lysosomal compartments through mechanisms that are not necessarily applicable to native γ-CD [272].

γ-CD-containing inclusion complexes represent a third category and should also be interpreted separately. In particular, the α-lipoic acid–γ-CD inclusion complex has been shown to activate adenosine monophosphate-activated protein kinase (AMPK) in the liver, a central energy-sensing enzyme regulated by the cellular AMP/ATP ratio. In animal models of type 2 diabetes, this complex reduced postprandial hyperglycemia and HbA1c levels, effects closely connected with broader metabolic regulation. However, these outcomes should be attributed to the formulation as a whole, in which γ-CD may enhance the stability, solubility, and bioactivity of α-lipoic acid, rather than to native γ-CD alone [273].

Similarly, Yoshikiyo et al. investigated γ-CD, perilla oil, and γ-CD–perilla oil inclusion complexes in a rat model characterized by elevated gastrointestinal 12-hydroxylated bile acid levels. Importantly, γ-CD administered alone aggravated liver injury and dyslipidemia, as reflected by increased AST, ALT, total cholesterol, and triglyceride levels relative to controls. In contrast, when γ-CD was administered as an inclusion complex with perilla oil, these unfavorable effects were attenuated, with reductions in liver injury markers and a trend toward improved lipid parameters compared with γ-CD alone. This observation is particularly important because it indicates that γ-CD should not be presented as uniformly beneficial across all metabolic contexts. Under conditions of disturbed bile acid homeostasis, γ-CD may interact with intestinal bile acids or lipid-derived metabolites in a manner that aggravates hepatic injury and dyslipidemia. Therefore, baseline metabolic status, bile acid composition, liver function, and co-administered lipids should be considered when evaluating γ-CD safety and efficacy. Future studies should specifically assess hepatic safety markers, bile acid profiles, and lipidomic changes before γ-CD can be proposed for nutritional or therapeutic use in populations with metabolic or hepatobiliary disorders [274].

Cho et al. investigated the effects of γ-CD supplementation in a murine model of diet-induced obesity and intestinal neoplasia. In mice fed a Western high-fat diet, γ-CD supplementation significantly reduced the size of intestinal adenomas and increased the proportion of smaller, less advanced lesions compared to the unsupplemented Western diet group. These effects were associated with increased apoptotic activity in intestinal tissues, suggesting a suppression of neoplastic progression under obese conditions. Importantly, γ-CD did not significantly alter body weight gain or food intake in obese mice, indicating that its effects were not mediated by reduced caloric intake but rather by metabolic or microbiota-related mechanisms. The authors proposed that γ-CD may be fermented in the colon to short-chain fatty acids, particularly butyrate, which is known to induce apoptosis in cells with dysregulated WNT/β-catenin signaling, a hallmark of colorectal tumorigenesis [275].

Zhu et al. investigated the effects of γ-cyclodextrin supplementation in mice fed a high-fat diet and reported that γ-CD exerted modest anti-obesity effects compared with other cyclodextrin types. In this model, γ-CD primarily reduced adipocyte diameter, without significantly decreasing relative fat mass. Histological analyses further showed that γ-CD attenuated lipid deposition in the liver, brown adipose tissue, and epididymal fat, although its effects were less pronounced than those observed for β-cyclodextrin. In the liver, γ-CD contributed to the reduction of macrovesicular and microvesicular steatosis, while in brown adipose tissue, it decreased lipid droplet enlargement induced by the high-fat diet. Overall, these findings suggest that γ-CD may confer partial, tissue-specific benefits in the regulation of lipid accumulation and adipose tissue morphology under high-fat dietary conditions, with effects that appear to be largely independent of caloric intake [269]. Zhang et al. developed a γ-CD-based inclusion complex with epigallocatechin-3-gallate (EGCG) and demonstrated its therapeutic efficacy in a murine model of ulcerative colitis. Beyond its anti-inflammatory activity, the γ-CD-containing formulation significantly reduced pro-inflammatory cytokines (TNF-α, IL-6, IL-1β) and oxidative stress markers, while restoring intestinal barrier-associated parameters. These effects are relevant in the context of metabolic disorders, as chronic intestinal inflammation and dysbiosis are closely linked to lipid metabolism disturbances and obesity-related complications. Notably, the γ-CD inclusion complex exhibited an enhanced capacity to modulate gut microbiota composition compared to EGCG alone, suggesting a role in regulating host metabolic homeostasis [276].

### 3.2. Effects of γ-CD on Glycemic Control and T2DM

A patent filed by Abbott Laboratories described the use of γ-CD as a functional ingredient capable of modulating postprandial metabolic responses. According to the disclosed data, oral administration of γ-CD was associated with a blunted postprandial glycemic response and reduced insulin secretion, despite its rapid digestion and absorption in the small intestine—a finding that contrasts with the expected behavior of readily metabolizable carbohydrates. The proposed mechanism suggests that γ-CD may influence glucose kinetics in a manner that attenuates glycemic excursions without inducing carbohydrate malabsorption or gastrointestinal adverse effects [277]. The interaction between γ-CD and α-amylase has been investigated, revealing that γ-CD can exert an inhibitory effect on enzyme activity under specific experimental conditions. The inhibitory effect was shown to be dependent on factors such as concentration, temperature, pH, and reaction time, with maximal inhibition observed at 10 mmol/L γ-CD, 45 °C, pH 5.9, and 120 min of incubation. Mechanistically, γ-CD was found to induce conformational changes in the secondary structure of α-amylase, including alterations in α-helix, β-sheet, and random coil content, suggesting a direct impact on protein folding and stability. In addition, spectroscopic analyses demonstrated changes in the enzyme’s intrinsic fluorescence, indicating modifications in the microenvironment of aromatic amino acid residues. Nuclear magnetic resonance data further supported the formation of a γ-CD–enzyme complex, in which part of the α-amylase molecule is accommodated within the hydrophobic cavity of γ-CD, while additional interactions are stabilized through hydrogen bonding at the external surface [278].

The effects of cyclodextrins on postprandial glycemic response have been evaluated in an experimental animal model using a real-time continuous glucose monitoring system. In this study, several cyclodextrin types, including α-, β-, γ-, and hydroxypropyl-β-cyclodextrin, were administered orally to rats at different doses. The findings demonstrated that in contrast to α- and β-cyclodextrins, γ-cyclodextrin significantly attenuated postprandial glycemic excursions following starch administration at both tested doses (10 and 100 mg/kg) [279].

γ-CD markedly reduced postprandial glycemia in a dose-dependent manner, with AUC decreases of 16.5% and 59.8% at 10 and 100 mg/kg, respectively. Male Zucker rats also exhibited lower glycemic spikes after γ-CD administration. These effects are attributed to the unique structural flexibility of γ-CD: its non-cylindrical, non-coplanar ring allows α-amylase to distort and hydrolyze the molecule, slowing glucose release and delaying absorption [280,281]. Unlike α- and β-CD, which are rigid and poorly hydrolyzed, γ-CD’s geometry facilitates enzymatic ring opening and controlled breakdown into maltose and glucose [282,283].

The data suggest that γ-CD holds potential for managing postprandial glycemia in prediabetes, type 2 diabetes, and other hyperglycemic conditions. It may also be applied in scenarios where high-carbohydrate diets are preferred, including gestational diabetes, reactive hypoglycemia, nocturnal hypoglycemia, and during prolonged exercise [281].

An in vivo study demonstrated that the combination of α-lipoic acid with γ-CD (αLA/γCD) significantly reduced HbA1c levels in KKAy mice compared to untreated controls, with a trend toward lower HbA1c than αLA alone, indicating a stronger hypoglycemic effect [284]. Plasma adiponectin levels tended to increase with αLA/γCD treatment, and PPARγ2 mRNA expression in adipose tissue was higher than with αLA alone, suggesting improved stability of αLA when complexed with γ-CD [285].

Furthermore, αLA/γCD significantly enhanced phosphorylation of AMPKα in the liver, consistent with the elevated adiponectin and PPARγ2 levels. Activation of AMPK is known to stimulate glucose utilization and fatty acid oxidation while inhibiting synthesis pathways, mimicking exercise-like metabolic effects [286,287,288]. Overall, these results indicate that γ-CD complexation enhances the antidiabetic properties of αLA, likely due to increased stability and bioavailability, with prior reports showing approximately 10% higher bioavailability of αLA/γCD compared to αLA alone [289].

## 4. Methods

**Figure 4:**
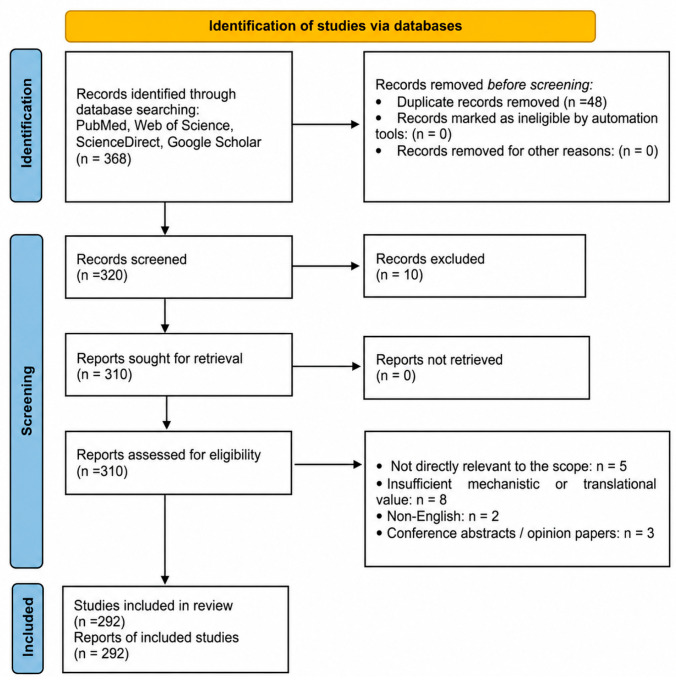
PRISMA flow diagram illustrating the literature search and study selection process for this review.

This review was carried out as a narrative overview with elements of a systematic search, guided by selected principles of the PRISMA (Preferred Reporting Items for Systematic Reviews and Meta-Analyses) framework. Searches were conducted across Google Scholar, PubMed, and ScienceDirect using combinations of the following terms: “*γ*-cyclodextrin,” “gut microbiota,” “prebiotic,” “short-chain fatty acids,” “lipids,” and “metabolic health.” Emphasis was placed on studies published within the last five years, especially the most recent year, while older studies were included if they provided foundational or unique insights. Articles were considered eligible if they addressed one or more of the following: (i) structural, chemical, or functional characteristics of *γ*-cyclodextrin; (ii) effects on gut microbiota composition and activity, including prebiotic potential; or (iii) influences on metabolic outcomes such as lipid metabolism, obesity, or type 2 diabetes. Only full-text articles in English were included, with abstracts, commentaries, and studies not directly relevant to the research focus being excluded. The objective of this review was to synthesize current knowledge and highlight key experimental evidence regarding *γ*-cyclodextrin and its potential role in modulating gut microbiota and metabolic health, rather than to perform a quantitative meta-analysis. The literature search and study selection process, following PRISMA principles, is illustrated in Figure 4.

## 5. Critical Appraisal of Current Evidence

The available evidence on γ-CD and metabolic regulation should be interpreted cautiously because it is heterogeneous in design, dose, biological model, and outcome assessment. In vitro fermentation studies provide useful mechanistic information regarding microbial utilization of γ-CD and SCFA production, but they cannot reproduce host absorption, immune signaling, bile acid metabolism, or enteroendocrine responses. Animal studies allow integrated assessment of microbiota–host interactions and metabolic outcomes, but their translational relevance is limited by species-specific microbiota composition, controlled diets, and doses that may not correspond to typical human exposure. Human data remain limited and are often derived from small cohorts or indirect endpoints, making it difficult to determine whether γ-CD has clinically meaningful effects on obesity, dyslipidemia, or glycemic control. Therefore, current findings support a plausible mechanistic role for γ-CD, but do not yet establish therapeutic efficacy.

## 6. Future Perspectives

Future research should aim to uncover how *γ*-cyclodextrins modulate gut microbial communities and their enzymes, with a focus on pathways that influence obesity, lipid metabolism, and type 2 diabetes. Understanding how *γ*-CDs and their inclusion complexes release bioactive compounds in the gut, and how they are metabolized by intestinal bacteria, will be critical for optimizing their metabolic effects. Multi-omics approaches—combining metagenomics, metabolomics, and transcriptomics—could clarify how *γ*-CDs impact short-chain fatty acid production, lipid handling, and glucose regulation, linking microbial changes to systemic metabolic outcomes.

In addition, future studies should include a systematic biological screening of native γ-CD and its main chemically modified derivatives. Hydroxypropylated, methylated, sulfobutylated, carboxymethylated, amino-functionalized, amphiphilic, and cross-linked γ-CD-based systems may differ substantially in solubility, charge, binding affinity, intestinal behavior, microbiota interaction, cellular uptake, and safety profile. Therefore, their biological effects should not be generalized from native γ-CD alone. Comparative screening should evaluate microbiota modulation, SCFA production, intestinal barrier function, lipid and bile acid metabolism, glucose homeostasis, inflammatory signaling, cytotoxicity, hemocompatibility, and hepatic or renal safety. Such an approach would support future structure–activity relationship analyses and guide the rational design of γ-CD-based formulations for nutritional, pharmaceutical, and metabolic applications (Table 5).

Advancing *γ*-CD-based interventions may allow targeted modulation of the microbiome to improve lipid profiles, reduce adiposity, and support glycemic control in prediabetes or diabetes. Integrating microbiome-informed strategies into the design of *γ*-CD formulations could enhance their prebiotic and metabolic benefits. Additionally, investigations into environmental, dietary, and epigenetic factors will be important for understanding variability in host responses. High-throughput sequencing and metabolite profiling may reveal new microbial species and pathways involved in obesity and dyslipidemia, while exploration of the gut–brain axis could open avenues for addressing metabolic dysfunction alongside neurological and inflammatory processes.

A novel perspective emerging from the current evidence is that γ-CD should not be considered only as an inert carrier or excipient but as a potential interface molecule linking food chemistry, microbial ecology, and host metabolic regulation. Its relevance may depend on whether inclusion complex formation modifies the delivery of bioactive compounds to the lower gut, whether γ-CD-derived fermentation selectively promotes SCFA-producing microbial networks, and whether these effects translate into measurable changes in inflammatory tone, insulin sensitivity, and lipid handling. This integrated view may help distinguish γ-CD from other cyclodextrins and provides a rationale for future microbiome-informed γ-CD formulations.

## 7. Conclusions

γ-CD is increasingly being investigated not only as a pharmaceutical and food excipient but also as a potential modulator of gut microbiota and host metabolic regulation. Current evidence suggests that γ-CD may influence microbial composition, SCFA production, lipid handling, inflammatory tone, and glycemic responses. However, these effects are context-dependent and vary according to cyclodextrin type, dose, dietary background, experimental model, and host metabolic status. The strongest evidence currently derives from in vitro and animal studies, whereas human data remain limited and insufficient to support definitive clinical recommendations.

From a clinical and nutritional perspective, γ-CD may represent a promising adjunctive strategy for modulating the gut–metabolism axis, particularly in conditions characterized by dysbiosis, low-grade inflammation, insulin resistance, and altered lipid metabolism. Nevertheless, its use for obesity, dyslipidemia, or T2DM cannot yet be considered established. Future research should prioritize randomized controlled human trials, standardized γ-CD formulations and doses, long-term safety assessment, multi-omics profiling, and direct comparison between native and modified cyclodextrins. Such studies are necessary to determine whether γ-CD can be translated into evidence-based nutritional or therapeutic interventions for metabolic disorders.

## Figures and Tables

**Table 1:** Nutritional, pharmaceutical, and biomedical applications of γ-CD inclusion complexes and their functional relevance.

Application Field	Guest Molecule	Cyclodextrin Type	Function/Benefit	Example
Pharmaceuticals	Poorly soluble drugs (e.g., antifungals, steroids)	γ-CD	Enhances solubility, bioavailability, and stability of drugs	Improved delivery of drugs, such as itraconazole and dexamethasone [39]
Food Industry	Flavors, essential oils, vitamins	γ-CD	Stabilizes volatile compounds, protects against oxidation, improves shelf life	Encapsulation of limonene (flavor) to prevent evaporation in citrus-based products [40]
Agriculture	Pesticides, herbicides, plant growth regulators	γ-CD	Improves solubility and stability of agrochemicals, reduces environmental toxicity	Cyclodextrin-enhanced delivery of herbicides, reducing runoff and improving soil uptake [41]
Biomedical	Anticancer agents, gene therapy vectors	γ-CD (including modified γ-CD)	Enhances targeted drug delivery, improves stability and controlled release of biomolecules	Cyclodextrin-based nanocarriers for delivering doxorubicin in cancer therapy [42]
Nanotechnology	Nanoparticles, drugs, bioactive molecules	γ-CD (including modified γ-CD)	Enables formation of nanocarriers for targeted delivery and diagnostic purposes	Cyclodextrin-based nanoparticles for targeted drug delivery in cancer treatment or imaging [43]

**Table 2:** Microbial changes in T2D and their clinical reference.

Taxonomic Level	Microbial Change in T2DM	Functional/Clinical Relevance	References
Phylum	↓ Firmicutes, ↑ Bacteroidetes and Proteobacteria	B/F ratio linked to elevated plasma glucose after oral glucose load	[233]
	↑ Firmicutes and Proteobacteria, ↓ Bacteroidetes	Enhanced F/B ratio observed in T2DM vs. non-diabetic; higher in complicated T2DM	[239,241]
Opportunistic pathogens	↑ *Bacteroides caccae*, *Clostridium hathewayi*, *Clostridium ramosum*, *Clostridium symbiosum*, *Eggerthella lenta*, *Escherichia coli*	Potential contribution to inflammation and dysbiosis	[242,243]
General with increased abundance	↑ *Blautia, Coprococcus*, *Sporobacter*, *Abiotrophia*, *Peptostreptococcus*, *Parasutterella*, *Collinsella*	Associated with metabolic imbalance	[242,243]
Butyrate-producing microbes (depleted)	↓ *Ruminococcus*, *Subdoligranulum*, *Eubacterium rectale*, *Faecalibacterium prausnitzii*, *Roseburia intestinalis, Roseburia inulinivorans*	Butyrate improves host homeostasis, insulin sensitivity, and reduces inflammation	[234,243]
Other depleted genera	↓ *Bacteroides*, *Prevotella*, *Bifidobacterium*	*Bifidobacterium* enhances gut barrier, lowers endotoxemia, reduces inflammation, and improves glucose tolerance	[244,245,246,247,248]
Lactobacillus species	↑ in European female T2DM cohort	Correlates with lower fasting glucose and improved HbA1c; unrelated to BMI	[239,245]
Protective species/genera	↑ *Akkermansia muciniphila*, *Faecalibacterium prausnitzii* (upon treatment/supplementation)	Maintains mucin layer integrity, reduces inflammation, improves insulin resistance and metabolic status	[251,252,253,254,255,256,257,258]
Pre-diabetic microbiota	↓ microbial diversity, ↓ *Akkermansia* and *Clostridium*, ↑ *Ruminococcus* and *Streptococcus*	Early dysbiosis may precede T2DM onset	[252]

Abbreviations: T2DM, type 2 diabetes mellitus; HbA1c, glycated hemoglobin; BMI, body mass index; F/B ratio, Firmicutes/Bacteroidetes ratio. ↑ indicates increased abundance or level; ↓ indicates decreased abundance or level.

**Table 3:** Dietary modulation of glycemic control and gut–metabolism interactions in T2DM: contextual evidence relevant to γ-CD research.

Study	Diet/Intervention	Main Glycemic Outcomes	Additional Findings	Ref.
Jian et al.	Low-energy diet	HbA1c ↓, FBG ↓, HOMA-IR ↓ (*p* < 0.001)	–	[253]
Ren et al.	Low-carbohydrate vs. low-fat diet	HbA1c ↓ in both; greater reduction in low-carb group (*p* < 0.01)	–	[254]
Candela et al.	Ma-Pi 2 diet vs. Italian Professional Association diet	FBG ↓ (*p* = 0.007), HOMA-IR ↓ (*p* = 0.0004); greater reductions in Ma-Pi 2 group	High-fiber diet improved insulin resistance	[255]
Ismael et al.	Mediterranean diet	HOMA-IR ↓ (−1.03 ± 2.64, *p* < 0.05), HbA1c ↓ (−0.67 ± 0.98, *p* < 0.05), FBG not significant	Cohen’s d: −0.41 for HOMA-IR, −0.70 for HbA1c	[256]
Deledda et al.	Ketogenic vs. Mediterranean diet	Ketogenic: HbA1c ↓ 1.1% (*p* = 0.012); Mediterranean: NS; FBG NS in both	–	[257]
Zhao et al.	High dietary fiber vs. Chinese Diabetes Society diet	HbA1c ↓ and FBG ↓ (*p* < 0.001); greater HbA1c reduction in fiber group from day 28 (−1.91 ± 0.24)	–	[258]
Chen et al.	High dietary fiber	HbA1c ↓, FBG ↓	–	[259]
Medina-Vera et al.	High fiber, low-energy diet	FFA ↓ 15.6%, HbA1c ↓ 7.2%	Functional food diet	[260]
Karusheva et al.	Reduced BCAA diet (BCAA−) vs. full amino acid diet (BCAA+)	Reduced insulin secretion, increased postprandial insulin sensitivity	–	[261]
Meleshko et al.	Personalized diet	Blood glucose ↓ (−2.36 ± 2.13 mmol/L, *p* < 0.05)	–	[262]
Shoer et al.	Personalized diet vs. Mediterranean diet	HbA1c better controlled in personalized diet	–	[263]

Abbreviations: HbA1c, glycated hemoglobin; FBG, fasting blood glucose; HOMA-IR, homeostatic model assessment of insulin resistance; FFA, free fatty acids; BCAA, branched-chain amino acids; NS, not statistically significant. Downward arrows (↓) denote a decrease in the indicated glycemic or metabolic parameter.

**Table 5:** Main γ-cyclodextrin derivatives and their potential relevance for biomedical and metabolic research.

γ-Cyclodextrin Form or Derivative	Structural/Functional Characteristic	Potential Relevance
Native γ-cyclodextrin	Natural cyclic oligosaccharide composed of eight glucose units; large internal cavity and high aqueous solubility	Inclusion complex formation, food and pharmaceutical applications, possible microbiota-mediated metabolic effects
Hydroxypropyl-γ-cyclodextrin	Hydroxypropyl substitution increases aqueous solubility and modifies interaction with lipophilic molecules	Drug solubilization, intracellular cholesterol trafficking studies, biomedical formulations
Methylated γ-cyclodextrin	Methyl substitution increases hydrophobic interactions and may alter membrane affinity	Enhanced complexation of hydrophobic guest molecules; requires careful safety evaluation
Sulfobutyl ether-γ-cyclodextrin	Anionic derivative with improved solubility and altered electrostatic interactions	Potential use in advanced drug delivery systems and charged guest molecule complexation
Carboxymethyl-γ-cyclodextrin	Anionic carboxymethylated derivative with modified solubility and binding properties	Controlled release systems and interaction with cationic molecules
Amino- or cationic γ-cyclodextrin derivatives	Positively charged derivatives obtained by amino-functionalization	Potential interaction with nucleic acids, negatively charged biomolecules, and targeted delivery systems
Amphiphilic γ-cyclodextrin derivatives	Derivatives containing hydrophobic substituents that promote self-assembly	Nanocarriers, micelle-like systems, and delivery of poorly soluble compounds
Cross-linked γ-cyclodextrin systems/γ-CD nanosponges	Polymeric networks formed through cross-linking of cyclodextrin units	Entrapment, stabilization, and controlled release of bioactive molecules
γ-CD-based metal–organic frameworks	Porous crystalline systems based on γ-CD and metal ions	Encapsulation, controlled delivery, and material-based biomedical applications
γ-CD inclusion complexes with bioactive compounds	Native or modified γ-CD complexed with guest molecules such as α-lipoic acid, oils, polyphenols, or drugs	Improved solubility, stability, bioavailability, and biological performance of guest molecules

## Data Availability

No new data were created or analyzed in this study. Data sharing is not applicable.
